# Carbon nanofibers (CNFs) supported cobalt- nickel sulfide (CoNi_2_S_4_) nanoparticles hybrid anode for high performance lithium ion capacitor

**DOI:** 10.1038/s41598-018-19787-z

**Published:** 2018-01-25

**Authors:** Ajay Jagadale, Xuan Zhou, Douglas Blaisdell, Sen Yang

**Affiliations:** 1 Department of Electrical and Computer Engineering, Kettering University, Flint, MI-48504 USA; 2 School of Science, MOE Key Laboratory for Nonequilibrium Synthesis and Modulation of Condensed Matter, Xi’an Jiaotong University, Xi’an, 710049 China

## Abstract

Lithium ion capacitors possess an ability to bridge the gap between lithium ion battery and supercapacitor. The main concern of fabricating lithium ion capacitors is poor rate capability and cyclic stability of the anode material which uses sluggish faradaic reactions to store an electric charge. Herein, we have fabricated high performance hybrid anode material based on carbon nanofibers (CNFs) and cobalt-nickel sulfide (CoNi_2_S_4_) nanoparticles via simple electrospinning and electrodeposition methods. Porous and high conducting CNF*@*CoNi_2_S_4_ electrode acts as an expressway network for electronic and ionic diffusion during charging-discharging processes. The effect of anode to cathode mass ratio on the performance has been studied by fabricating lithium ion capacitors with different mass ratios. The surface controlled contribution of CNF*@*CoNi_2_S_4_ electrode was 73% which demonstrates its excellent rate capability. Lithium ion capacitor fabricated with CNF*@*CoNi_2_S_4_ to AC mass ratio of 1:2.6 showed excellent energy density of 85.4 Wh kg^−1^ with the power density of 150 W kg^−1^. Also, even at the high power density of 15 kW kg^−1^, the cell provided the energy density of 35 Wh kg^−1^. This work offers a new strategy for designing high-performance hybrid anode with the combination of simple and cost effective approaches.

## Introduction

High energy density, high power density and long cycle life are the main requirements of future energy storage devices in order to use them in different moderns applications such as consumer electronic devices, hybrid electric vehicles and large scale grid energy storage^1^. In the last two decades, number of research articles have been published in the field of energy storage devices which are mainly focusing on the supercapacitors (SCs) and lithium ion batteries (LIBs). SCs store an electrical energy via non-faradaic process which includes adsorption/desorption of electrolytic charges on the surface of electrode materials. However, LIBs use intercalation/deintercalation of lithium ions in the host materials. Due to their electric double layer type (EDL) charge storage mechanism, SCs provide high power density (>10 kW kg^−1^) and good cyclic life (>10^5^ cycles), however, due to the limited accumulation of charges they exhibit lower energy performance (<10 Wh kg^−1^). Besides, LIBs deliver high energy performance (150–200 Wh kg^−1^) due to diffusion controlled slow faradaic reactions throughout the active material. In the present state of art, these two devices are inadequate to be used for many applications such as electric vehicles and plugged in electric vehicles.

Recently, Lithium ion capacitors (LICs) have attracted great attention because of their extraordinary electrochemical properties. LICs bridge the gap between SCs and LIBs by combining merits of both systems^2^. Basically, LICs are made up of high energy LIB anode, high power SC cathode and Li ion containing organic electrolyte (mainly LIB electrolyte). In 2001, Amatucci *et al*.^3^ first fabricated LIC at the Telcordia technologies using nanostructured Li_4_Ti_5_O_12_ (LTO) anode, activated carbon (AC) cathode and Li^+^ ion containing organic electrolyte. Till date, many research articles have been published which are mainly focused on the novel anode and cathode materials. Generally, Li^+^ insertion based anode provides high energy density to the LIC, however, due to the sluggish kinetics, it results into poor power performance. In order to improve both power and energy densities simultaneously, preparation of nanostructured and high electrical conductivity anodes are desirable. Previously, research in the anode materials have been focused on the development of hybrid electrodes by applying conductive carbon coating and preparing controlled nanostructured morphology. Carbon coating on the active material surface provides fast electronic diffusion which leads to the increased rate capability and high power performance. The nanostructured morphology offer reduced pathways for ionic and electronic diffusion and enhanced surface activity for charge storage. Previously, different anode materials have been reported such as LTO^4^, graphite^5^, graphdiyne^6^, Si^7^, Mn_3_O_4_-graphene^8^, TiO_2_^9^, graphene^10^, LiTi_1_._5_Zr_0_._5_(PO_4_)_3_^11^, soft carbon^12^, NbN^13^, LiMnBO_3_^14^, SnO_2_-C^15^, hard carbon^16^, Nb_2_O_5_*@*graphene^17^, TiO_2_-B^18^, etc. Cathodes such as AC, Nb_2_CT_x_-carbon nanotube^19^, graphene^20^, carbide-derived carbon^21^ have been mostly reported. In the aforementioned materials, binders and conductive additives are preferably used. However, these components increase the dead mass of the electrode that makes an obstacle for ionic diffusion in the active material which further deteriorates the cell performance. Also, anodes like LTO, TiO_2_ and Nb_2_O_5_ possess poor electrical conductivity that restricts them to be efficiently utilized in LIC. In this scenario, preparation of self-standing binder-free high conductivity electrodes is highly anticipated.

Recently, transition metal sulfide (TMS) anodes have attracted great attention because of their superior capacity values as compared to the conventional graphite (372 mAh g^−1^) and LTO (170 mAh g^−1^) anodes. Also, the high electrical conductivity of TMSs facilitates pathways for electron transportation which lead to the high power performance. Recently, very few sulfides have been utilized as an anode in LIC, for example, Zhang *et al*.^22^ fabricated MoS_2_ incorporated onto 3D porous graphene and Amaresh *et al*.^23^ used cubic CoS_2_ nanoparticles as anodes for LIC. Apart from this, different TMSs have been frequently reported as anodes in LIBs including WS_2_^24^, MoS_2_^25^, Cu_2_S^26^, Ni_3_S_4_ and NiS_1_._03_^27^, SnS_2_^28^, CoS^29^, Bi_2_S_3_^30^, NiCo_2_S_4_^31^, etc. Among the available sulfide anodes, mixed metal sulfides, for example, NiCo_2_S_4_ and CoNi_2_S_4_ have great importance due to their excellent electrical conductivity, high redox activity and high theoretical capacity around 703 mAh g^−1^.

On the other hand, carbon matrix, especially carbon nanofibers can enhance the electrical conductivity of the electrode and endure the stresses occurred during cycling^32,33^. By considering above facts, in the present investigation, we have prepared carbon nanofibers (CNF) using electrospinning method and used as a scaffold for the electrodeposition of cobalt nickel sulfide (CoNi_2_S_4_) nanoparticles. This nanoparticle coated carbon nanofiber hybrid webs provide high surface area and open channels for efficient electron/ion transport which leads to the superior electrochemical performance. Furthermore, LICs were prepared by using CNF*@*CoNi_2_S_4_ as an anode and activated carbon as a cathode. AC//CNF*@*CoNi_2_S_4_ LIC fabricated with anode to cathode mass ratio of 1:2.6 delivered energy density of 85.4 Wh kg^−1^ with the power density of 150 W kg^−1^ and showed excellent cyclic stability of 96% after 5000 cycles.

## Results and Discussion

**Figure 1:**
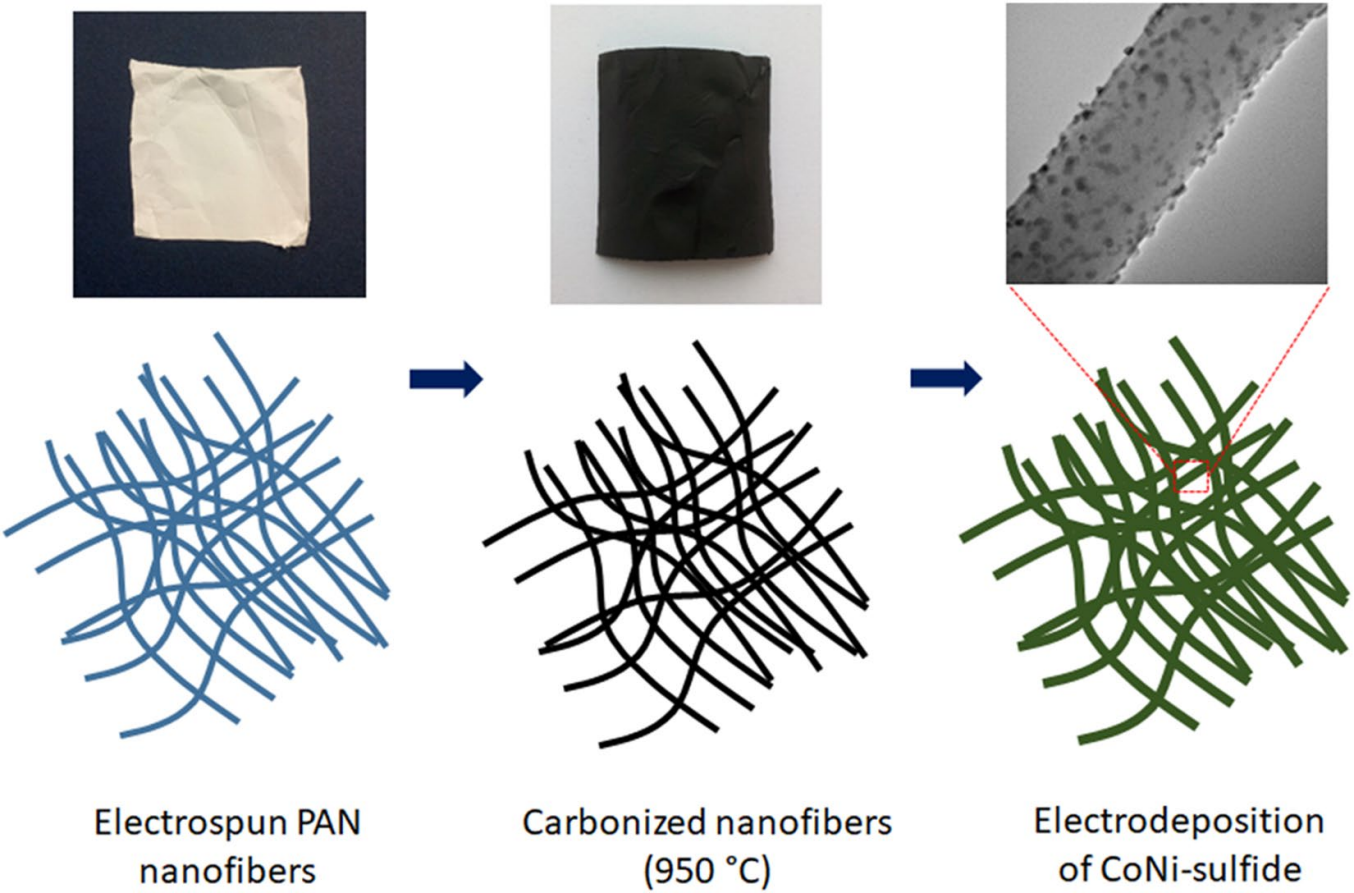
schematics of the formation process of CNF*@*CoNi_2_S_4_ electrode.

Figure 1 shows schematic of formation process of CNF*@*CoNi_2_S_4_ anode which involves three steps. In the first step, PAN nanofibers were prepared using electrospinning method. Furthermore, as synthesized PAN nanofibers were stabilized and carbonized. Finally, carbonized nanofibers were used as a substrate for the deposition of Co-Ni sulfides. As shown in the figure, CoNi_2_S_4_ nanoparticles were homogeneously deposited on the surface of CNF.

**Figure 2:**
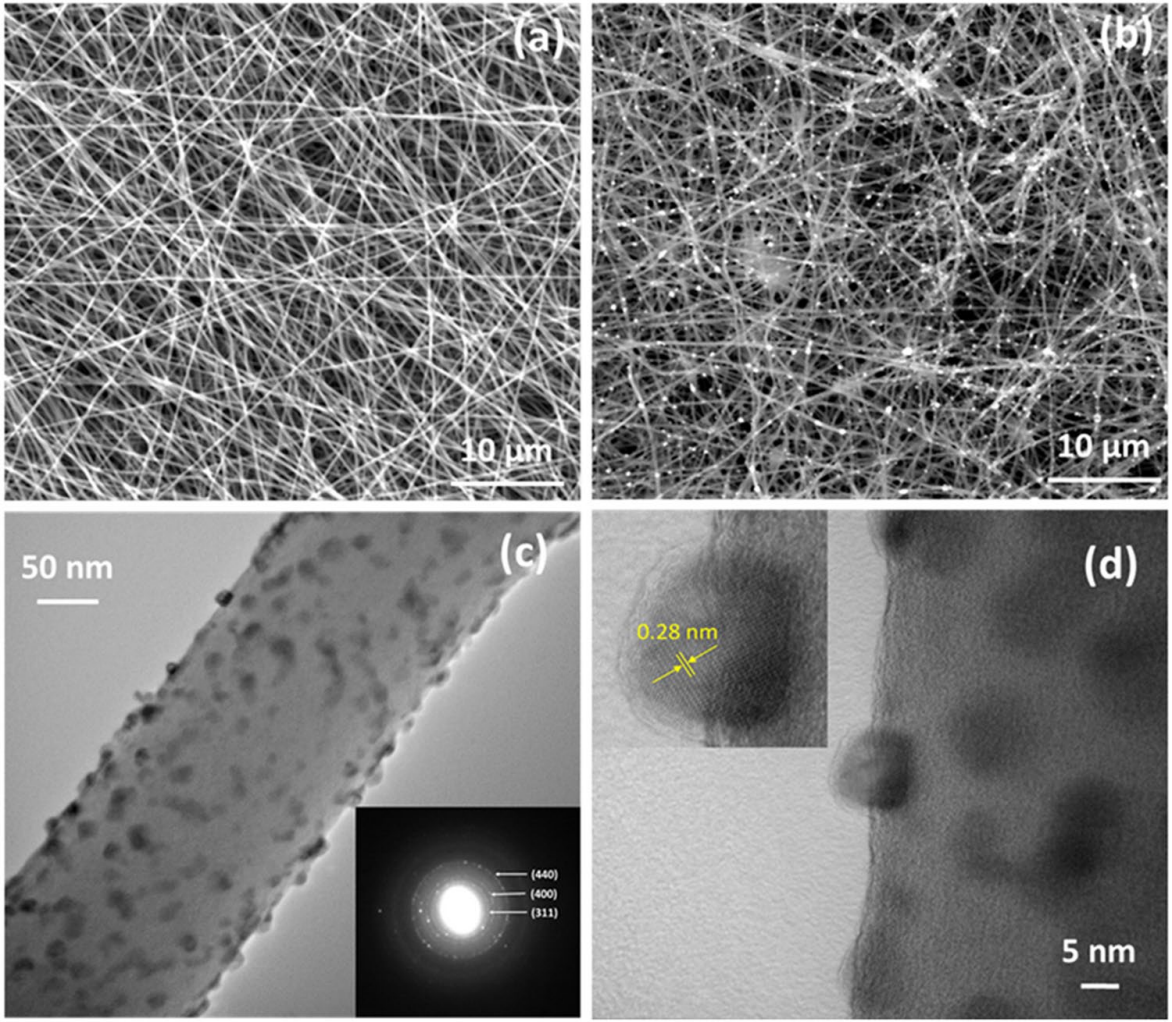
E-SEM images of (**a**) bare CNFs, (**b**) CNFs loaded with CoNi_2_S_4_ nanoparticles, (**c**) TEM image of single CNF decorated with CoNi_2_S_4_ nanoparticles (Inset: SAED pattern), and (**d**) high resolution-TEM image of CoNi_2_S_4_ nanoparticle supported on CNF (Inset: lattice spacing corresponds to 311 plane of single CoNi_2_S_4_ nanoparticle).

Morphology of the CNF*@*CoNi_2_S_4_ is investigated by E-SEM and TEM. Figure 2a,b show E-SEM images of bare CNFs and CNFs loaded with CoNi_2_S_4_ samples. From the figure, it is seen that the bare carbon nanofibers construct 3D porous structure with randomly dispersed smooth fibers of the diameter of 200 to 300 nm. As shown in the Fig. 2b, CoNi_2_S_4_ particles are formed on the surface of CNFs, however, these are microsized larger particles. In the electrodeposition method, particles start growing at electrochemically active sites, some particles grow faster than normal because of inhomogeneous distribution of surface activity. In order to visualize the very surface of the CNF, TEM has been used. Figure 2c shows TEM image of the CNF*@*CoNi_2_S_4_ sample which confirms the formation of CoNi_2_S_4_ nanoparticles on the surface of CNF with the diameter of 10 to 15 nm. The SAED pattern depicts the polycrystalline nature of CoNi_2_S_4_ by indexing planes as (311), (400) and (440) (Inset of Fig. 2c). Figure 2d shows high resolution TEM image of the CoNi_2_S_4_ particles which depicts that the nanoparticles are firmly attached to the surface of CNF (inset of Fig. 2d). Interestingly, it seems that the particles are hard to be detached since there were no particles observed in the background of TEM images. Also, the inset of the Fig. 2d shows that the lattice spacing of 0.28 nm corresponds to the 311 crystal planes of CoNi_2_S_4_ which is consistent with XRD results. Recently, similar structure has been reported by Li *et al*.^34^ by fabricating Co_3_O_4_ nanoparticles-decorated carbon nanofibers as an air-cathode for rechargeable Zn-air batteries. In another study, Shen *et al*.^35^ have loaded Tin nanoparticles on the carbon nanofibers for their application as an anode in LIBs.

**Figure 3:**
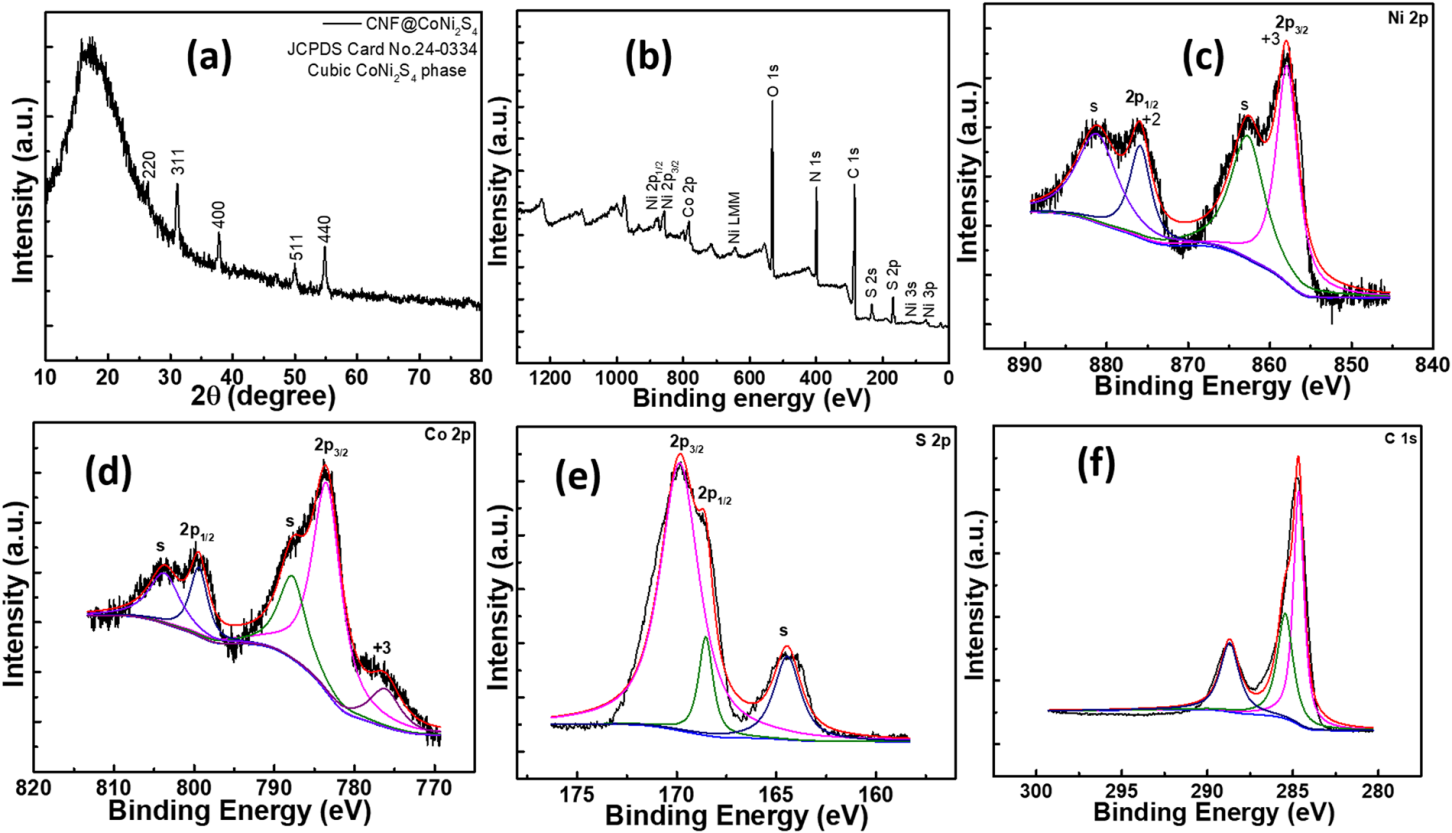
(**a**) XRD pattern, (**b**) XPS survey, (**c**) Ni 2p, (**d**) Co 2p, (**e**) S 2p, and (**f**) C 1 s spectra of CNF*@*CoNi_2_S_4_ sample.

Figure 3a shows XRD pattern of CNF*@*CoNi_2_S_4_ sample which represents diffraction peaks at 2θ of 26.3, 31.2, 37.8, 50.0 and 54.8° can be indexed to (220), (311), (400), (511) and (440) planes of cubic CoNi_2_S_4_ (JCPDS card no. 24–0334). Peaks from the carbon nanofibers are not observed due to their amorphous nature. Also, the average crystallite size of CoNi_2_S_4_ was estimated as 25.7 nm on the basis of full width at half maxima intensity of XRD peak for (311) plane using Scherrer’s formula,

1 $$D=\frac{0.89\lambda }{\beta \,\cos \,\theta }$$

where ‘D’ is average crystallite size, ‘β’ is full width at half maxima, ‘λ’ is wavelength of X-ray used and ‘θ’ is diffraction angle. This size is reasonably comparable with the size observed with high resolution TEM image. This XRD data is quite consistent with the literature^35,36^. Furthermore, the XPS measurements were carried out in order to understand the surface electronic states of the CNF*@*CoNi_2_S_4_ sample. Figure 3b shows the survey spectrum recorded for CNF*@*CoNi_2_S_4_ sample which demonstrates the presence of Ni, Co, S and C elements. The high resolution spectrum of Ni 2p is fitted with two shakeup satellites (s) by using a Gaussian fitting method. As shown in the Fig. 3c, two strong peaks centered at binding energies of 857.8 and 875.8 eV are attributed to Ni^3+^ and Ni^2+^ ions^37^. The satellite peaks at around 862.7 and 881.1 eV are two shakeup type peaks of Ni at the upper binding energy sides of Ni 2p_3/2_ and Ni 2p_1/2_, respectively^38^. Figure 3d shows the Co 2p spectrum, the binding energy centered at 776.2 eV of the Co 2p peak are assigned to Co^3+^ and the binding energies at 783.4 and 799.4 eV to Co^2+^. The existence of Co 2p_3/2_ and Co 2p_1/2_ peaks indicates the presence of both Co^3+^ and Co^2+^ ions^39^. As shown in Fig. 3e, the S 2p spectrum depicts two peaks with the binding energies centered at 169.8 and 164.4 eV which correspond to the main and shakeup satellite peaks, respectively. The second satellite peak designates the bonding between metal and sulfur, in the present case, Ni-S and Co-S bondings^37^. As the CNF*@*CoNi_2_S_4_ electrode was mainly formed of carbon, it was desirable to understand the electronic states of the carbon. Figure 3f shows the C1s spectrum comprised of three different peaks centered at 284.6, 285.4 and 288.6 eV which might be attributed to the single and double bonds between carbon (C-C, C=C), carbon interacted with hydroxyl and epoxy groups (C-OH, C-O-C), and carboxylic groups (COO), respectively^40^. This result confirms the formation of CoNi_2_S_4_ on the surface of CNF and also supports the data obtained by XRD study.

Figure S1 (supporting information) shows 1^st^, 2^nd^, and 3^rd^ CV curves of CNF*@*CoNi_2_S_4_, 1^st^ CV cycle depicts three distinct cathodic peaks at 1.7, 1.2 and 0.7 V which can be assigned to the reduction of Co^2+^ and Ni^3+^ into metallic Co and Ni, respectively. Also, peaks observed at around 2.0, 2.2 and 2.4 V in the subsequent anodic scan can be attributed to the oxidation of metallic Ni and Co to NiS_x_ and CoS_x_, respectively^31,41^. In the 2^nd^ and 3^rd^ cycles, the main cathodic peaks shifted toward a higher potential and the anodic peaks shifted slightly toward lower potential. This shifting and decrease in the cathodic peak currents might be due to the existence of certain irreversible reactions associated with the formation of a solid electrolyte interphase (SEI) film on the electrode surface^42^. Furthermore, the overlapping of 2^nd^ and 3^rd^ CV curves indicates an excellent reversibility of the CNF*@*CoNi_2_S_4_ electrode. The redox reversibility of the CNF*@*CoNi_2_S_4_ electrode has been evaluated using Laviron’s theory with the calculation of heterogeneous electron transfer rate constant using Kochi’s method (supporting information, S1). The electrochemical reaction associated with the charging-discharging process of the CNF*@*CoNi_2_S_4_ electrode can be illustrated as follows^41,43^,

2 $${{\rm{CoNi}}}_{{\rm{2}}}{{\rm{S}}}_{{\rm{4}}}+{{\rm{8Li}}}^{+}+{{\rm{8e}}}^{-}\to {\rm{Co}}+{\rm{2Ni}}+{{\rm{4Li}}}_{{\rm{2}}}{\rm{S}}$$

3 $${\rm{Co}}+{{\rm{Li}}}_{{\rm{2}}}{\rm{S}}\leftrightarrow \,\mathrm{CoS}\,+{{\rm{2Li}}}^{+}+{{\rm{2e}}}^{-}$$

4 $${\rm{Ni}}+{{\rm{Li}}}_{{\rm{2}}}{\rm{S}}\leftrightarrow {\rm{NiS}}+{{\rm{2Li}}}^{+}+{{\rm{2e}}}^{-}$$

5 $$\mathrm{CoS}\,+{1/\mathrm{3Li}}_{{\rm{2}}}{\rm{S}}\leftrightarrow {1/\mathrm{3Co}}_{{\rm{3}}}{{\rm{S}}}_{{\rm{4}}}+{2/\mathrm{3Li}}^{+}+{2/\mathrm{3e}}^{-}$$

**Figure 4:**
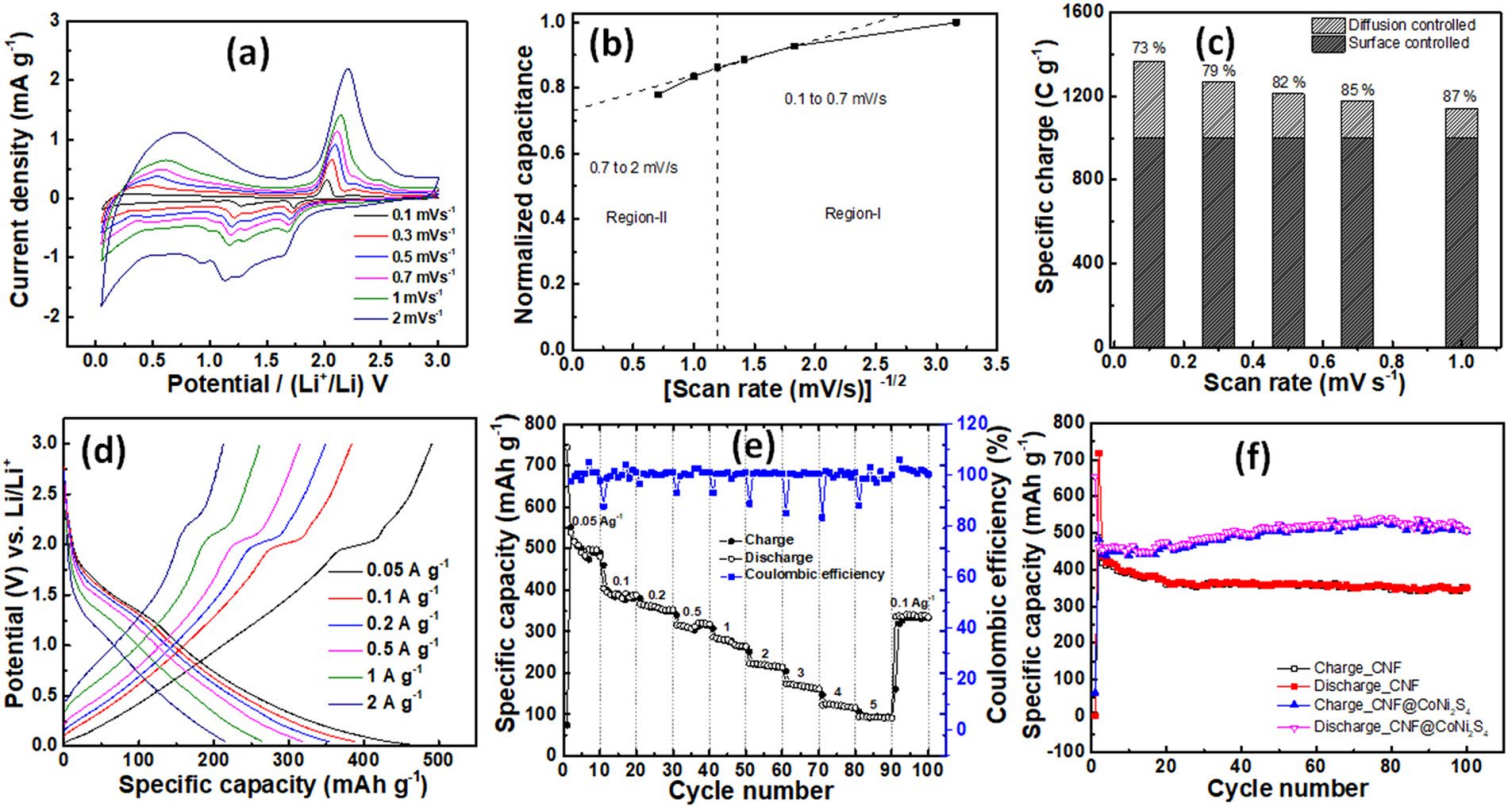
(**a**) CV curves of CNF*@*CoNi_2_S_4_ electrode at different scan rates range from 0.1 to 2 mV s^−1^, (**b**) variation of normalized capacitance and [scan rate (mV s^−1^)]^−1/2^, (**c**) surface and diffusion controlled charge storage contributions at different scan rates for CNF*@*CoNi_2_S_4_ electrode, (**d**) GCD curves of of CNF*@*CoNi_2_S_4_ electrode at different current densities range from 0.05–2 A g^−1^, (**e**) rate perfromance of CNF*@*CoNi_2_S_4_ at current densities of 0.05, 0.1, 0.2, 0.5, 1, 2, 3, 4, 5 A g^−1^, and (**f**) cyclic performance of CNF*@*CoNi_2_S_4_ and CNF at the current density of 0.1 A g^−1^.

In order to investigate the electrochemical performance of CNF*@*CoNi_2_S_4_ electrode, CV measurements were carried out at various scan rates ranging from 0.1 to 2 mV s^−1^ (Fig. 4a). As shown from CV curves, when the scan rate increased, area under the curve also increased which is in accordance with the high rate capability of the CNF*@*CoNi_2_S_4_ electrode. Also, Fig. S3 (supporting information) shows the variation of specific charge and scan rate. The capacity retention of 78% at relatively highest scan rate (2 mV s^−1^) demonstrates high rate capability of the CNF*@*CoNi_2_S_4_ electrode. Furthermore, it is necessary to ensure the quantitative analysis of charge storage contributions in the anode materials, recently, Trasatti procedure has attracted great attention because of its ability to distinguish different charge storage contributions in the anode materials^44–46^. According to the Trasatti procedure, the total charge storage (q*) involves both outer (q_o_) and inner (q_i_) charge storage, this can be seen by the equation below,

6 $$q\ast ={q}_{0}+{q}_{i}$$

The outer charge is mainly originated due to the surface capacitive reactions and the inner charge due to the diffusion controlled insertion reaction. Therefore, specific charge storage at various scan rates can be estimated by using equation,

7 $$q={q}_{\infty }+k/{v}^{1/2}$$

where *v* and *k* are scan rate and constant, respectively. Figure 4b shows the plot of normalized capacitance versus inverse of root of scan rate (*v*^−1/2^) for CNF*@*CoNi_2_S_4_ from a scan rate of 0.1–2 mV s^−1^. The plot distinguishes into two regions, the region-I (0.1–0.7 mV s^−1^) and the region –II (0.7–2 mV s^−1^). In the region-I, the normalized capacitance was seen to be independent or inadequately dependent on the scan rate, however, in the region-II, normalized capacitance started to decrease quickly when the scan rate increased. The capacitive or the surface controlled contribution can be estimated by extrapolated y-intercept which was found to be 73%. Here, it is worth mentioning that the remaining only 27% contribution was originated from the diffusion controlled kinetics. In order to prepare excellent anode materials for LIC, it is desirable to use electrode with greater capacitive or surface-controlled contributions. This kind of electrodes can facilitate excellent rate capability or the power capability without compromising energy performance. Recently, the capacitive contributions for 3D interconnected TiC and 2D nanosheets structured ZnMn_2_O_4_-graphene have been reported as 78% (0.5 mV s^−1^) and 24.2% (0.2 mV s^−1^), respectively^47,48^. An excellent capacitive contribution in the present case might be attributed to the excellent rate capability of the electrode which can be originated due to the high conductivity and open porous structure of the CNF*@*CoNi_2_S_4_ electrode. Furthermore, as shown in the Fig. 4c, when the scan rate increased from 0.1 to 1 mV s^−1^, the diffusion controlled contribution decreased which can be attributed to the diffusion limitations and increased ohmic contribution^48^.

The rate capability was examined by recording galvanostatic charging discharging (GCD) curves of the CNF*@*CoNi_2_S_4_ electrode at various current densities ranging from 0.05–2 A g^−1^. Figure 4d shows the GCD curves at various current densities performed within the potential window of 0.005–3 V vs. (Li^+^/Li), which depicts the excellent coulombic efficiency of 100%. As shown in the Fig. 4e, CNF*@*CoNi_2_S_4_ was able to deliver a specific capacity of 497 mAh g^−1^ at 0.05 A g^−1^ (10^th^ cycle), which is far greater than the theoretical capacities of graphite and LTO^21^. Also, when the current density increased by 10 folds to 0.5 A g^−1^, the specific capacity was found to be 319 mAh g^−1^. The rate capability in the present case is quite comparable with that of obtained for carbon paper supported CoNi_2_S_4_ nanorods arrays^49^. This is because, good conducting, porous and high surface area CNFs facilitate fast and efficient ionic and electronic diffusion during charging discharging process. Furthermore, after the rigorous cycling at higher current density of 10 A g^−1^, the specific capacity at the 0.1 A g^−1^ was recovered back close to initial cycling capacity, which certainly proves the superior rate capability of the CNF*@*CoNi_2_S_4_ electrode.

Furthermore, in order to understand the cyclic performance of the CNF*@*CoNi_2_S_4_ electrode, the charge-discharge cycling was performed at the current density of 0.1 Ag^−1^ for 100 cycles and compared with the bare CNF (Fig. 4f). During cycling, the capacity of the CNF*@*CoNi_2_S_4_ increased up to certain extent and appeared to be stabilized. However, the capacity of the bare carbon nanofibers was decreased during the first couple of cycles and stabilized. The capacity increment in case of CNF*@*CoNi_2_S_4_ can be attributed to the electrochemical activation of the electrode^50^. An excellent electrochemical performance in terms of specific capacity, rate capability and cyclic stability of the CNF*@*CoNi_2_S_4_ electrode can be attributed to the following points. 1) Well-distributed nanoparticles of the CoNi_2_S_4_ on the CNF surface facilitate enhanced surface area for the electrochemical charge storage, besides, the extra spacing between two adjacent particles offers extra space for the expansion of the particles during lithiation process which improves cyclic performance. Also, as discussed in the TEM results, these particles are strongly attached to the CNF which also empowers the cyclic life of the particle-derived nanocomposite electrodes. 2) The structure of CNF*@*CoNi_2_S_4_ electrode is mainly comprised of CNF webs and CoNi_2_S_4_ nanoparticles which is highly porous structure that offers reduced pathways for electrolytic diffusion, moreover, the good conducting behavior of both CoNi_2_S_4_ and CNF acts as an expressway for electronic diffusion through the electrode network, this enhances the rate capability of the electrode. 3) Carbon matrix near the interface of nanoparticle and the CNF accommodates the expansion of the CoNi_2_S_4_ and prevents the pulverization of nanoparticles which further improves cyclic life of the electrode.

**Figure 5:**
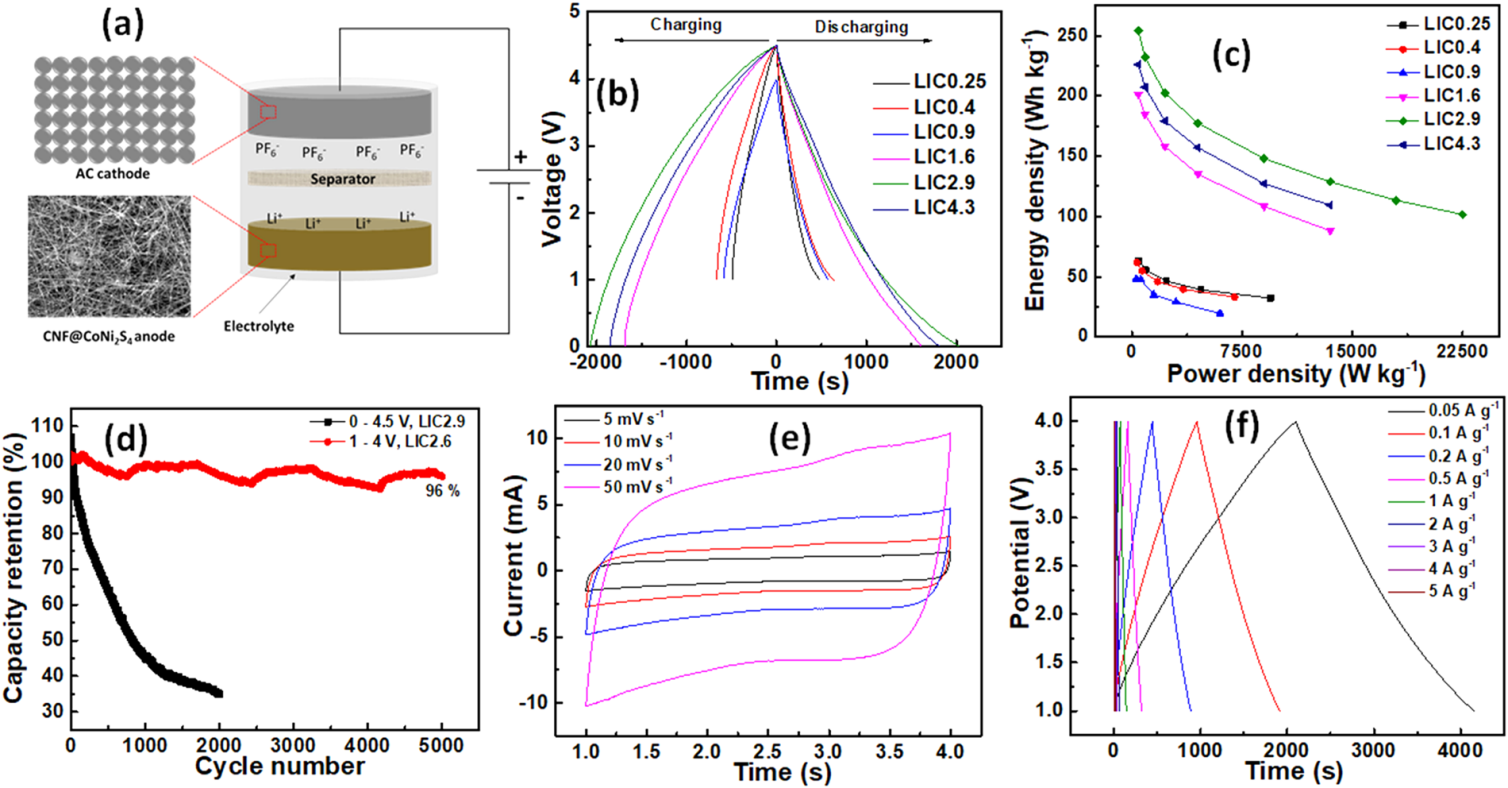
(**a**) Schematic of the LIC based on AC cathode and CNF*@*CoNi_2_S_4_ anode, (**b**) GCD curves of LICs fabricated with different anode to cathode mass ratios at constant current density of 0.1 A g^−1^, (**c**) Ragone plot with values of energy and power densities for different LICs, (**d**) cyclic performance of LIC2.6 (1–4 V) and LIC2.9 (0–4.5 V) at the current density of 2 A g^−1^, (**e**) CV curves of LIC2.6 at different scan rates range from 5–50 mV s^−1^, and (**f**) GCD curves of LIC2.6 at different current densities range from 0.05–5 A g^−1^.

As shown in the Fig. 5a, LICs were fabricated using AC as a cathode and prelithiated CNF*@*CoNi_2_S_4_ as an anode in 1 M LiPF_6_ in EC-DEC electrolyte solution. Before fabrication of the LICs, CNF*@*CoNi_2_S_4_ electrodes were first prelithiated by directly contacting with lithium foil with one or two drops of electrolyte. The charge storage mechanism of the LIC is based on both faradaic and non-faradaic electrochemical reactions. During charging process, Li^+^ ions from the electrolyte are intercalated into the CNF*@*CoNi_2_S_4_ nanocomposite material, while ${{\rm{PF}}}_{{\rm{6}}}^{-}$ ions are adsorbed on the high surface area AC cathode. In order to utilize full benefits of CNF*@*CoNi_2_S_4_ electrode, it is necessary to balance charges on both electrodes. In the present investigation, different LICs were fabricated with anode to cathode mass ratios of 1:0.25, 1:0.4, 1:0.9, 1:1.6, 1:2.9 and 1:4.3, and respectively labeled as LIC0.25, LIC0.4, LIC0.9, LIC1.6, LIC2.9 and LIC4.3. Furthermore, the electrochemical performance of the LICs was examined using cyclic voltammetry, GCD and EIS. Figure 5b shows the GCD curves of LICs fabricated with different anode to cathode ratios at 0.1 A g^−1^. Interestingly, the GCD curves are slightly deviated from the ideal triangular shape which might be due to the combination of both faradaic and non-faradaic reactions involved during charging-discharging processes. Also, GCD curves are symmetric in nature which suggests its high reversibility and excellent coulombic efficiency. The operating potential window of the each LIC was optimized and found to be 1 to 4.5 V (LIC0.25 and LIC0.4), 1 to 4 V (LIC0.9) and 0 to 4.5 V (LIC1.6, LIC2.9 and LIC4.3). The capacitance (C_cell_) of the cell was calculated using equation ${C}_{cell}=i\times t/{\rm{\Delta }}V$, where *i* is the discharging current (A), *t* is the discharging time (s) and *ΔV* is a difference between upper and lower potentials of the discharging curve (V). Further, the specific capacitance (C_SP_) of the cell was calculated based on the total mass of both electrodes by using a formula ${C}_{SP}=4{C}_{cell}/m$, where m is the total mass (g). The maximum specific capacitances (at 0.1 A g^−1^) were obtained as 74, 72, 77, 143, 181 and 161 F g^−1^ for LIC0.25, LIC0.4, LIC0.9, LIC1.6, LIC2.9 and LIC4.3, respectively.

Furthermore, the energy and power densities of the LICs were calculated using formulae given in the experimental section. Figure S3 shows GCD curves for all LICs at different current densities (supporting information). Figure 5c shows Ragone plots composed of values of energy and power densities for LICs fabricated with different anode to cathode ratios. It is worth noting that the energy and power densities of LIC fabricated with anode to cathode mass ratio of 1:2.9 found to be phenomenally high as 254 Wh kg^−1^ at the power rating of 450 W kg^−1^. Also, the same LIC can facilitate the maximum power density of 22.5 kW kg^−1^ with energy density of 101 Wh kg^−1^. These values of energy and power densities are still beyond the limit of supercapacitor and LIB, respectively. Interestingly, the LICs with lower mass ratios showed poor values of energy and power densities which might be attributed to the imbalanced charge storage on both electrodes which minimizes an operating potential window of the cell. Further, when the mass ratio increased, the energy and power densities were also increased up to certain extent, however, when the mass ratio increased around 1:4.3, the energy and power densities were decreased because of the increased inactive or dead masses in the cathode material. Furthermore, due to the excellent capacitive performance of LIC2.9, it was employed for the cyclic stability study by GCD cycling for 2000 cycles at 2 A g^−1^ within potential window of 0.005–4.5 V. As shown in the Fig. 5d, the capacity of the LIC2.9 was surprising decreased which might be due to the large potential window that causes decomposition of electrolyte and deprivation of cycling stability. In order to confirm the interpretation on the capacity loss, we fabricated the new LIC with approximately similar anode to cathode mass ratio of 1:2.6 (LIC2.6) and employed for stability study at current density of 2 A g^−1^ within a reduced potential window of 1–4 V. The stability performance of LIC2.6 has been showed in the Fig. 5d which demonstrated the huge capacity retention of 96% after 5000 cycles. Figure 5e shows the cyclic voltammetry curves recorded for the LIC2.6 at different scan rates ranging from 5–50 mV s^−1^. The curves are rather deviated from the ideal rectangular shape which can be attributed to the combination of both faradaic and non-faradaic electrochemical processes on both electrodes. Also, when the scan rate increased, the area under the curves also increased which is in good agreement with ideal supercapacitive behavior, also it confirms that the electrochemical reactions are surface controlled rather than diffusion controlled. Figure 5f shows the GCD curve of LIC2.6 at different current densities varied from 0.05–5 A g^−1^ within a potential window of 1–4 V. GCD curves are triangular with little bit of deviation from the ideal triangular shape which is consistent with the cyclic voltammetry study (Fig. 5e). The values of specific capacitance were found to be 137, 127, 118, 106, 95, 82, 71, 63 and 56 F g^−1^ at the current densities of 0.05, 0.1, 0.2, 0.5, 1, 2, 3, 4 and 5 A g^−1^, respectively. When the current density increased by 100 folds to 5 A g^−1^, the value of the specific capacitance still maintained up to 41% which demonstrates the superior rate capability of the LIC2.6.

**Figure 6:**
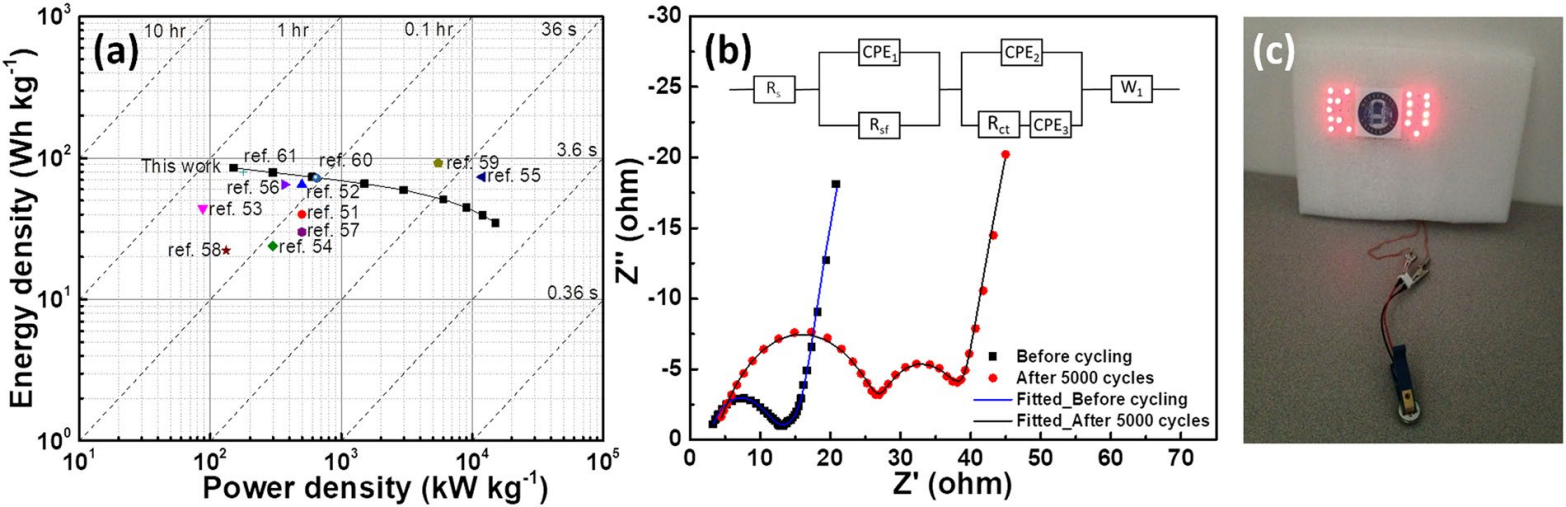
(**a**) Ragone plot of LIC2.6 with scattered points designate the data of LICs taken from the literature, (**b**) Nyquist plots of LIC2.6 before and after cycling within a frequency range from 0.1 Hz to 100 kHz at the open circuit potential (Inset: equivalent circuit fitted for the impedance data), and (**c**) A demonstration of 18 LEDs lighted by the AC//CNF*@*CoNi_2_S_4_ LIC.

Figure 6a shows the Ragone plot for LIC2.6 obtained by the calculation of energy and power densities from the GCD curves. LIC2.6 showed the maximum energy density of the 85.4 Wh kg^−1^ with the power delivery of 150 W kg^−1^ at the current density of 0.05 A g^−1^. Interestingly, even at the high power density of 15 kW kg^−1^, LIC2.6 still can maintain the energy density of 35 Wh kg^−1^. These values of energy and power densities in the present case are quite comparable with the literature^51–61^.

In order to understand the effect of cycling on the resistive behavior of the LIC, LIC2.6 was employed for EIS analysis. Figure 6b shows Nyquist plots of LIC2.6 obtained before cycling and after 5000 cycles (2 A g^−1^) within a frequency range from 0.1 Hz to 100 kHz for an applied AC amplitude of 5 mV at the open circuit potential. As shown in the Nyquist plot, two semicircles are observed at high to mid-frequency region with inclined line at low frequency region, however, second semicircle is not distinct in the plot of before cycling. The equivalent circuit model fitted for the impedance data is shown in the inset of Fig. 6b and the calculated data is plotted as a line plots. The equivalent circuit exhibits elements such as R_s_, R_sf_, R_ct_, CPE and W_1_ which correspond to the solution resistance, lithium ion migration resistance through the SEI (solid electrolyte interphase) layer^62^, charge transfer resistance, constant phase element and Warburg impedance, respectively. An intercept to the real Z axis designates as the solution resistance Rs which can be caused due to the ionic resistance of Celgard separator, electrical resistance of both AC and CNF*@*CoNi_2_S_4_ and contact resistances between active materials and current collectors. In the present case, R_s_ was estimated to be 2.6 and 3.5 Ω for before cycling and after cycling, respectively. It is seen that the internal resistance of the cell doesn’t change significantly. The first semicircle in the high to mid-frequency region can be recognized as the SEI film resistance which might be formed on the negative electrode of the LIC and the values were found to be 3.3 and 9.7 Ω for before and after cycling. Interestingly, after cycling, the SEI film resistance increased which can be attributed to the increased thickness of the SEI layer over cycling^63^. The second semicircle in the mid-frequency region represents the charge transfer resistance which was estimated to be 9 and 24.8 Ω for before and after cycling, respectively. The increased value of the R_ct_ is ascribed to the increased contact resistance between active materials and current collector^64^. The capacitive component in the equivalent circuit is substituted with CPE because of the porous nature of both AC and CNF*@*CoNi_2_S_4_ electrodes. Warburg impedance W1 is associated with the diffusion of lithium ions through the network of CNF*@*CoNi_2_S_4_ anode. Summarized fitting parameters estimated for the LIC2.6 are shown in the Table 1.

**Table 1:** Fitting EIS parameters obtained from the LIC cell before and after 5000 cycles.

Sample	R_s_ (Ω)	R_sf_ (Ω)	R_ct_ (Ω)	C (F)
Before cycling	2.6	3.3	9.0	1.3 × 10^−3^
After cycling	3.5	9.7	24.8	1.6 × 10^−3^

In order to demonstrate the practical applicability of the LIC, we charged LIC2.6 at contact potential of 3 V for less than 60 s and discharged through 18 LEDs making KU (initials of Kettering University) which lasts for 2 min as shown in the Fig. 6c. This indicates its potential application in future appliances.

## Conclusions

In summary, we have fabricated a novel LIC based on CNF supported CoNi_2_S_4_ nanoparticles anode and AC cathode in Li ions containing nonaqueous electrolyte in order to bridge the gap between LIB and supercapacitor. The CNF webs decorated with CoNi_2_S_4_ nanoparticles offer highly porous structure which reduces pathways for the electrolytic diffusion, while, the good conducting behavior of both CoNi_2_S_4_ and CNF acts as an expressway for electronic diffusion for efficient electrochemical reactions at the electrode/electrolyte interface. The capacitive or the surface controlled contribution of CNF*@*CoNi_2_S_4_ electrode was found to be 73% which demonstrates excellent rate capability and ensures its ability to be used for LIC. LIC2.6 fabricated with CNF*@*CoNi_2_S_4_ to AC mass ratio of 1:2.6 showed excellent energy density of 85.4 Wh kg^−1^ with the power density of 150 W kg^−1^. Also, even at the huge power density of 15 kW kg^−1^, LIC2.6 can supply the energy density of 35 Wh kg^−1^. Therefore, it is worth mentioning that the CNF*@*CoNi_2_S_4_ electrode is an excellent anode material for the fabrication of high performance LICs to be used for electric and plugged in electric vehicles.

## Methods

### Chemicals

Cobalt chloride (CoCl_2_·6H_2_O) and nickel chloride (NiCl_2_·6H_2_O) were purchased from Alfa Aesar. Polyacrylonitrile (PAN, Mw = 150,000) and Thiourea were purchased from Sigma-Aldrich.

### Synthesis of CNF*@*CoNi_2_S_4_ hybrid anode

At first carbon nanofibers were fabricated via electrospinning method. Briefly, homogeneous solution of 12% PAN in DMF was prepared and filled into a syringe with a needle having an inner diameter of 0.64 mm. The electrospinning process was done at an applied voltage of 14 kV with a feeding speed of 1 mL h^−1^ and the distance between the tip of the needle and the collector was 20 cm. As prepared PAN fibers were pre-oxidized at 280 °C for 2 h with a heating rate of 1 °C min^−1^. Furthermore, pre-oxidized fibers were employed for carbonization process at 950 °C for 0.5 h with a heating rate of 3 °C min^−1^ under Ar atmosphere. After carbonization, CNF mats were cut into suitable pieces and employed as a substrate for electrodeposition process. Prior to deposition, CNF mats were first electrochemically treated in 1 M H_2_SO_4_ solution using cyclic voltammetry within the potential window of −0.7 to +1.2 V vs. Ag/AgCl at the scan rate of 50 mV s^−1^ for 100 cycles. Co-Ni sulfide was coated on the CNF mats using potentiodynamic electrodeposition method within a potential window of +0.2 to −1.2 V vs. Ag/AgCl at the scan rate of 5 mV s^−1^ for 25 cycles. An electrochemical cell was assembled in a three-electrode configuration in which Cu supported CNF mat, Pt and Ag/AgCl were used as working, counter and reference electrodes, respectively. The electrodeposition bath was formed of 5 mM CoCl_2_.6H_2_O, 10 mM NiCl_2_.6H_2_O and 0.75 M thiourea in 50 ml deionized (DI) water. After deposition, electrodes were thoroughly rinsed with DI water and dried at room temperature. In order to improve the crystallinity of the sample, dried electrodes were further heat treated at 300 °C for 2 h with a heating rate of 5 °C min^−1^ under Ar atmosphere. These electrodes were punched into circular discs and weighted via microbalance with an accuracy of 0.01 mg.

### Material characterization

The morphology of the sample was characterized by environmental scanning electron microscope, E-SEM, (Quanta 200 s, Phillips Electron Optics Company) and transmission electron microscope, TEM, (JEOL JEM-2100, USA). X-ray diffraction, XRD pattern was collected using a diffractometer (Rigaku SmartLab) equipped with a Cu Kα radiation source (λ = 1.5406 A). The electronic states of the different elements in the sample were examined using X-ray photoelectron spectroscopy, XPS, (Kratos Axis Ultra).

### Electrochemical measurement

All electrochemical measurements were performed at room temperature. The galvanostatic charge-discharge was performed on a LAND-CT2001A battery testing system. The cyclic voltammetry (CV) and electrochemical impedance spectroscopy (EIS) measurements were made through Biologic SP-300 potentiostat. Electrochemical performance of the CNF*@*CoNi_2_S_4_ electrode was evaluated in half coin cells (CR2032) assembled in an Ar-filled glove box. Lithium metal was used as a counter and reference electrode and Celgard membrane was used as a separator. The electrolyte was prepared by dissolving 1 M LiPF_6_ in the mixture of ethylene carbonate (EC) and diethyl carbonate (DEC) with a volume ratio of 1:2. The LICs such as LIC0.25, LIC0.4, LIC0.9, LIC1.6, LIC2.9, LIC2.6 and LIC4.3 were formed of CNF*@*CoNi_2_S_4_ electrode masses as 4.4, 3.9, 3.2, 2.9, 1.7, 1.4 and 1.5 mg cm^−2^, and the electrode densities as 0.09, 0.08, 0.06, 0.06, 0.03, 0.03 and 0.03 g cm^−3^ respectively. Also, LIC was fabricated with prelithiated CNF*@*CoNi_2_S_4_ as anode, AC as a cathode and Celgard membrane as a separator. Prelithiation of the CNF*@*CoNi_2_S_4_ was performed by making direct contact of Li metal with one or two drops of electrolyte in between for 3h^48,65^. AC cathode was fabricated by mixing AC powder, acetylene black and polyvinylidene fluoride (PVDF) in N-methyl-2-pyrrolidone (NMP) solvent with a mass ratio of 80:10:10 and the slurry was coated on the Al foil. Further, the electrodes were dried at 120 °C for 8 h. The energy and power density of the LIC were calculated using following formulae,

8 $$P=I\times {\rm{\Delta }}V/m$$

9 $$E=P\times t/3600$$

10 $${\rm{\Delta }}V={V}_{{\rm{\max }}}-{V}_{{\rm{\min }}}$$

Where, *I* is the discharge current (A), *t* is the discharge time (s), *m* is the total active material on both electrodes (kg) and *V*_*max*_ and *V*_*min*_ are the upper and lower potentials during charge-discharge process.

### Data Availability

The datasets generated during the current study are available from the corresponding author on reasonable request.

## Electronic supplementary material

Supplementary Information
