# Supplementary material for: Carbon nanofibers (CNFs) supported cobalt- nickel sulfide (CoNi2S4) nanoparticles hybrid anode for high performance lithium ion capacitor
Source: Sci Rep. 2018 Jan 25;8:1602. doi: 10.1038/s41598-018-19787-z (PMC5785478; doi:10.1038/s41598-018-19787-z)
Supplement: Supplementary file 1 — Supplementary Information [file 41598_2018_19787_MOESM1_ESM.doc]

**Supporting information**

**Carbon nanofibers (CNFs) supported cobalt- nickel sulfide (CoNi_2_S_4_) nanoparticles hybrid anode for high performance lithium ion capacitor.**

Ajay Jagadale^a^, Xuan Zhou^a^*, Douglas Blaisdell^a^, Sen Yang^b^

*^a^ Department of Electrical and Computer Engineering, Kettering University, Flint, MI-48504, USA.*

*^b^ School of Science, MOE Key Laboratory for Nonequilibrium Synthesis and Modulation of Condensed Matter, Xi'an Jiaotong University, Xi'an 710049, China*

**Fig. S1** first three CV curves of CNF@CoNi_2_S_4_ electrode at the scan rate of 0.1 mV s^–1^.

**Fig. S2** variation of peak currents and scanning rates.

**Fig. S3** variation of specific charge calculated at different scan rates and scan rate of CNF@CoNi_2_S_4_ electrode.


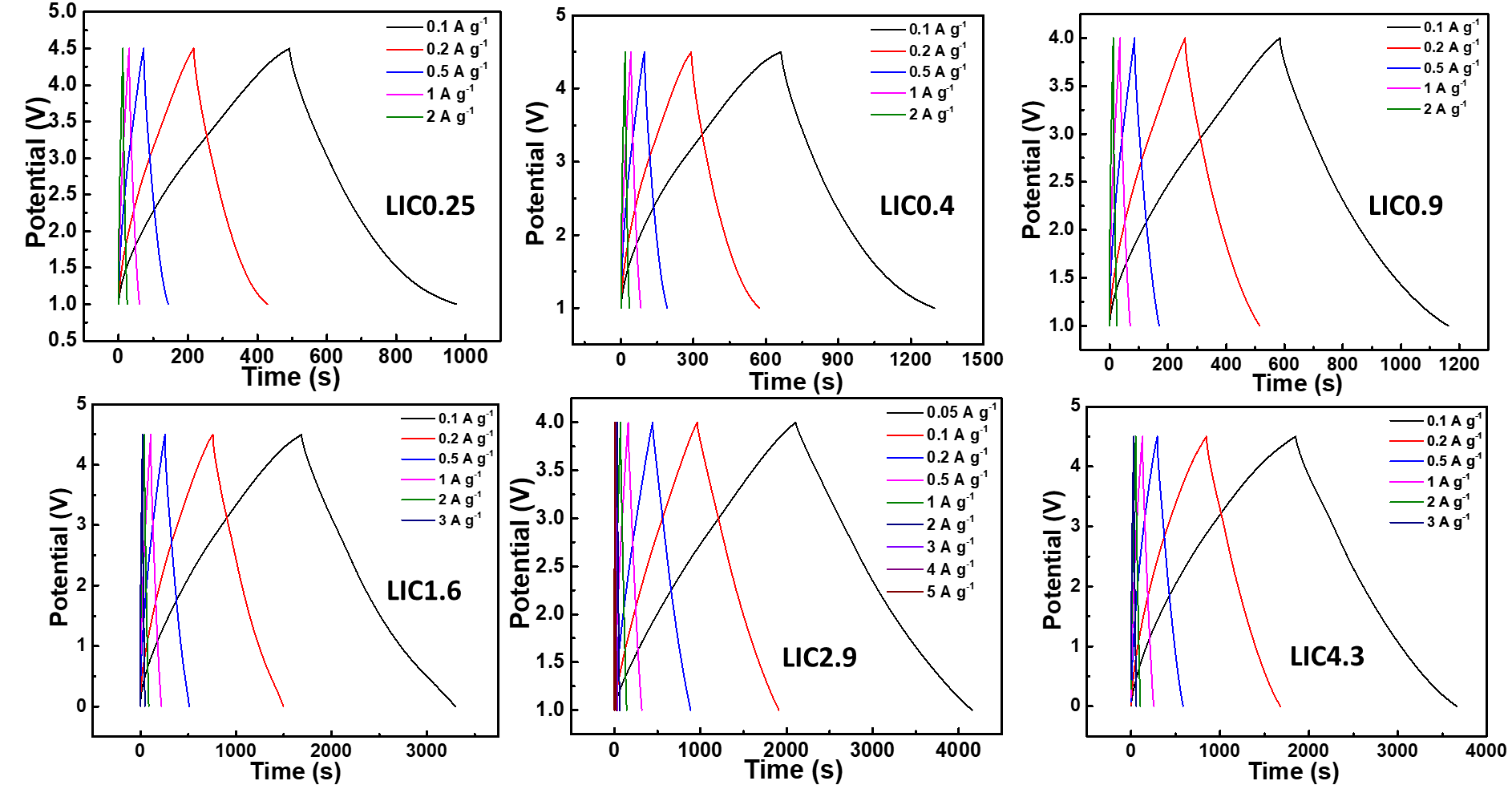


**Fig. S4** GCD curves at different current densities of LIC0.25, LIC0.4, LIC0.9, LIC1.6, LIC2.9, and LIC4.3.

**S1. Reversibility of CNF@CoNi_2_S_4_**

Reversibility of oxidation/reduction of CNF@CoNi_2_S_4_ was figured out by means of cyclic voltammetry at the scan rate of 0.0001V/s. Cyclic voltammograms of CNF@CoNi_2_S_4_ are given in the fig. S1. From the figure, CNF@CoNi_2_S_4_ exhibited cathodic peak at Ec = 1.73V and anodic peak at Ea = 2.03 V. Hence, ΔE = Ec – Ea = -0.3. This value indicates that the reversibility of the electrode is good. The heterogeneous electron transfer rate constant (k^0^) of CNF@CoNi_2_S_4_ electrode was estimated using Kochi’s method ^1^,

$k^{0}=2.18\left[\frac{\alpha vnFD_{0}}{RT}\right]^{1/2}\exp\left[-\frac{\alpha^{2}nF\Delta E_{p}}{RT}\right]$ (1)

Where, ‘α’ is an electron transfer coefficient, n is the number of electrons involved into redox process (n = 8), D_0_ is the diffusion coefficient which was obtained as 2.73× 10^−10^ cm^2^ s^−1^ (calculation of diffusion coefficient is given below), F is Faraday constant (96,485 C mol^−1^), ‘*v’* is scan rate (0.0001Vs^−1^), R is a gas constant (8.314 J mol^−1^ K^−1^), T is a temperature in K (T = 293K).

**S2. Calculation of diffusion coefficient**

The peak currents in cathodic and anodic scans at different scanning rates are employed to calculate the Li-ion diffusion coefficient. CV curves from the fig. 4a of main manuscript have been considered. Fig S2 shows the variation of anodic and cathodic peak currents and scan rates. The diffusion coefficients of Li ions in CNF@CoNi_2_S_4_ were calculated based on the Randles–Sevchik equation ^2^,

$I_{p}=0.4463\left(\frac{F^{3}}{RT}\right)^{1/2}n^{3/2}AD^{1/2}C_{0}v^{1/2}$ (2)

where ‘I_p_’ is the peak current (A), ‘R’ is the gas constant (8.314 J mol^−1^ K^−1^), T (K) is the absolute temperature, ‘F’ is the Faraday constant (96500 C mol^−1^), ‘n’ is the number of electrons transferred per molecule (8), ‘A’ is the active surface area of the electrode (0.56 cm^2^), C_0_ is the concentration of Li ions in the electrolyte (1.0×10^−3^ mol cm^−3^), D is the Li^+^ ion diffusion coefficient (cm^2^ s^−1^), and ‘v’ is the scanning rate (V s^−1^). From the slopes of the fitting lines collected from peak currents in the fig. S2, the apparent diffusion coefficients, D, of CNF@CoNi_2_S_4_ are 4.63×10^−10^ and 8.42×10^−11^ cm^2^ s^−1^ for anodic and cathodic processes, respectively. For the above calculation of k^0^, the average value of diffusion coefficient has been taken as 2.73×10^−10^ cm^2^ s^−1^.

**References**

1 German, N., Ramanavicius, A. & Ramanaviciene, A. Electrochemical deposition of gold nanoparticles on graphite rod for glucose biosensing. *Sensors and Actuators B: Chemical* **203**, 25-34 (2014).

2 Liu, C., Zhang, C., Fu, H., Nan, X. & Cao, G. Exploiting High‐Performance Anode through Tuning the Character of Chemical Bonds for Li‐Ion Batteries and Capacitors. *Advanced Energy Materials* **7** (2017).
